# Low-Glycemic-Index Foods Can Decrease Systolic and Diastolic Blood Pressure in the Short Term

**DOI:** 10.1155/2015/801268

**Published:** 2015-10-05

**Authors:** Mina Hosseininasab, Abdolreza Norouzy, Mohsen Nematy, Shokoufeh Bonakdaran

**Affiliations:** ^1^Nutrition Research Center and Department of Nutrition, School of Medicine, Mashhad University of Medical Sciences, Mashhad 91779-48564, Iran; ^2^Endocrinology and Metabolism Center, Ghaem Hospital, Iran

## Abstract

*Background*. We aimed to compare the effects of low- and high-GI foods on 24-hour ambulatory blood pressure.* Methods*. This longitudinal study was performed on 30 women, aged 18 to 40 years, during 24 hours. In the first leg of study all recruited subjects were assigned to LGI period for 24 hours and, after a 2-week washout period, all subjects were assigned to HGI period. BP was measured every hour during the 24-hour monitoring.* Results*. After the intervention, there were significant decreases in SBP and DBP in the LGI period (102.26 ± 14.18 mmHg versus 112.86 ± 9.33 mmHg for SBP and 66.96 ± 10.39 mmHg versus 74.46 ± 7.61 mmHg for DBP) (*P* = 0.00 and *P* = 0.002, resp.). However, in the HGI period, there was no significant change in SBP or DBP (110.66 ± 9.85 versus 111.80 ± 9.57 for SBP and 71.16 ± 9.16 versus 74.26 ± 10.09 for DBP) (*P* = 0.6 and *P* = 0.06, resp.).* Conclusion*. The results suggest that LGI foods may be beneficial in reducing 24-hour BP.

## 1. Introduction

High blood pressure (BP) is defined as systolic BP (SBP) ≥ 140 mmHg and/or diastolic BP (DBP) ≥ 90 mmHg. High BP is an independent risk factor for cardiovascular diseases, stroke, and kidney diseases. It is also one of the most common health problems worldwide [1].

As previous studies indicate, several dietary factors such as increased salt intake, insufficient potassium, obesity, overweight, excess alcohol intake, and high consumption of carbohydrates (CHO) including sugars and soft drinks can increase BP [2, 3]. However, total CHO intake has not been consistently associated with either increased or decreased BP. Differences in the type and source of dietary CHO may have various impacts on the relationship between CHO intake and BP [4].

Prevention of elevated BP is an important public health issue with the aim of reducing the overall disease rate, caused by hypertension [1]. In fact, reduced BP could have significant impacts on cardiovascular diseases, morbidity, and mortality [5].

Glycemic index (GI) provides a numeric classification of CHO foods, based on their glycemic response that reflects the rise in postprandial glycemia [6]. As previous studies have revealed, changes in SBP and DBP are associated with glycemic load (GL) and GI [3, 7, 8]. For instance, Philippou et al. in a study performed in 2009 found that a 6-month intensive lifestyle modification including dietary GI manipulation, in addition to healthy eating and weight loss, affects arterial compliance and 24-hour BP, which are risk factors for coronary heart disease (CHD). Low-GI (LGI) food has been suggested to be more effective in reducing CHD risks including pulse wave velocity and 24-hour BP [6].

However, previous studies have not considered factors such as obesity and family history of hypertension [3, 6, 8, 9]. Also, since participants differed from nonparticipants in terms of characteristics such as age, weight, height, ethnicity, and body mass index (BMI), the possibility of selection bias, which limits the generalization of the results, has not been ruled out.

In the present study, we hypothesized that 24-hour LGI foods would significantly decrease 24-hour ambulatory BP. The aim of our study is assessing the effect of changing GI of foods on 24-hour BP.

## 2. Materials and Methods

### 2.1. Subjects

In this longitudinal study, subjects' demographic information including age, gender, weight, and height was gathered before the study. Overall, 30 women, aged 18–40 years, were selected based on BP level (below 140/90 mmHg).

The inclusion criteria were as follows: (1) SBP < 140 mmHg; (2) DBP < 90 mmHg; (3) nonuse of medications during the intervention; and (4) no drug therapy for hypertension.

The exclusion criteria were as follows: (1) diabetes; (2) prior history of diseases affecting BP (e.g., renal and cardiac diseases); (3) pregnancy and/or lactation; (4) vigorous physical activity during the intervention; (5) smoking; and (6) BP traces that were missing >4 hourly means over the 24 h.

All procedures involving human subjects were approved by the Research Ethics Committee of Mashhad University of Medical Sciences. Written informed consents were obtained from all the subjects. A checklist including demographic data and questions related to the inclusion criteria was completed by all participants at baseline.

### 2.2. Study Procedure

In the first leg of study all recruited subjects were assigned to LGI period for 24 hours and, after a 2-week washout period, all subjects were assigned to HGI period. The subjects were asked to only consume the determined foods. The participants maintained their usual diet and lifestyle during a washout period. A dietitian counseled the participants during 24 hours of intervention to ensure adherence to diets. The subjects were controlled in an isolated location and monitored by the dietitian for 24 hours. The designated foods were consumed by the subjects at the determined hours. Also, full and timely consumption of foods was controlled by the dietitian.

In our study, the energy intake of diets was similar in the two groups (1900–2000 k cal). Also, macronutrient distribution was equivalently prescribed in the two groups (75–80% CHO, 8-9% proteins, and 12–15% fat). The amounts of fat and protein in the diets were below the standard recommended levels since our study focused on foods rich in CHO for a better analysis of the effect of CHO on BP.

GI values were extracted from the International Tables of GI and GL and Values reference scale based on GI glucose = 100 [7]. Dietary GL was calculated as the product of dietary GI and CHO intake divided by 100. The daily dietary GL of each subject was calculated and summed up, and the GI of the whole diet was calculated, using the following formula (see [3]):

(1) dietary  GLtotal  available  CHO  intake  in  the  day×100%.

Tables 1 and 2 show details and ingredients of consumed foods in 24-hour LGI and HGI periods. Total 24-hour dietary GL of each subject was 34.1 gr for LGI period and 132 gr for HGI period. The daily dietary GI of each subject was 42.76% for LGI period and 84.46% for HGI period.

Fasting blood samples (after 12 hours of fasting and avoiding alcohol and exercise for 24 hours) were obtained at baseline to exclude diabetic cases from the study.

### 2.3. BP Screening

For screening BP, a cuff was fitted to the participants' nondominant arm by a trained nurse and removed after 24 hours. BP and heart rate (HR) were measured every hour during the 24-hour monitoring, providing a total of 24 readings within 24 hours. Subjects were instructed to immobilize their arms during cuff inflation. A wrist stabilizer was used to support the arm to ensure the best possible positioning of the device and minimize movements.

The patients were instructed to follow their routine daily activities and avoid any vigorous exercises, alcohol use, smoking, and use of medications while wearing the cuff. BP traces that were missing more than 4 hourly means over the 24 hours were excluded from the analysis.

### 2.4. Statistical Analysis

Statistical analyses were performed by SPSS version 11.5. First, Kolmogorov-Smirnov test was performed to assess the normality of quantitative variables. Data were presented as mean ± SD. Paired *t*-test was used for comparing variables before and after the intervention in each group and within groups. *P* values less than 0.05 were considered statistically significant.

## 3. Results

The current study was conducted on 30 female subjects, with the mean age of 24.63 ± 3.20 years (minimum of 18 and maximum of 32 years), mean weight of 57.16 ± 9.07 kg (minimum of 39 and maximum of 75 kg), mean height of 162.83 ± 6.11 cm (minimum of 150 and maximum of 178 cm), and BMI of 21.47 ± 2.60 kg/m^2^ (minimum of 17.26 and maximum of 27.55 kg/m^2^).

Table 3 shows the baseline characteristics of study subjects. According to this table, most of the participants (53.3%) were within the age range of 20–25 years. Overall, 6.7% of participants were underweight (BMI < 18.5 kg/m^2^), 83.3% had a normal weight (BMI = 18.5–24.9 kg/m^2^), and 10% were overweight (BMI = 25–29.9 kg/m^2^); none of the participants were obese (BMI < 30 kg/m^2^).

### 3.1. Blood Pressure (BP)

The total values of 24-hour SBP and DBP in the LGI group were 73,559 mmHg and 47,390 mmHg, respectively. Also, the total values of 24-hour SBP and DBP in the HGI group were 73,546 mmHg and 47,929 mmHg, respectively. These numbers are the sum of mean blood pressure readings for each time point.

Data analysis showed that both dietary plans resulted in reduced SBP and DBP after the intervention, although only changes in the LGI period were significant (*P* = 0.001 and *P* = 0.002, resp.). In fact, in the HGI period, there was no significant change in SBP or DBP (*P* = 0.6 and *P* = 0.06, resp.) (Table 4).

After analysis divided by day and night (overnight rested and fasted) showed reduces in SBP and DBP over night were significant only in the LGI period (*P* = 0.01 and *P* = 0.02) (Table 5).

**Figure 1:**
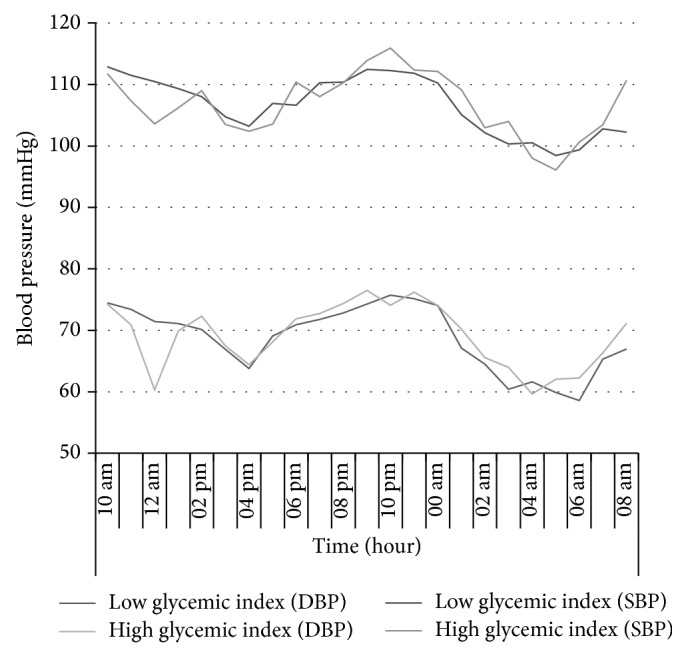
Constant SBP and DBP measurements in HGI and LGI groups at baseline and after the 24-hour interventions (values are expressed as mean ± SEM).

BP analysis showed no significant differences in the mean and changes of SBP or DBP between LGI and HGI period during 24 hours (*P* = 0.89 and *P* = 0.31, resp.) but since SBP and DBP were constantly evaluated during day and night, significant differences were observed between the two periods at night (*P* = 0.01 and *P* = 0.04) (Table 6). The area under the curve was 2344.4 for SBP and 1508.89 for DBP in the LGI period. Also, the area under the curve was 2344.03 for SBP and 1516.08 for DBP in the HGI period (Figure 1).

### 3.2. Heart Rate (HR)

We analyzed the relationship between HR and changes in dietary GI and GL intakes.

There was a significant decrease in HR after the intervention in the two periods (*P* = 0.02 and *P* = 0.01, resp.) (Table 4). But after analysis divided by day and night (overnight rested and fasted) there was only significant reduce in HR in LGI period (Table 5).

However, the analysis of HR showed no significant difference in 24-hour mean and changes of HR between LGI and HGI periods (*P* = 0.76) (Table 6).

## 4. Discussion

In the present longitudinal study, we examined the effects of dietary GI and GL changes on BP and HR. This study demonstrated that LGI foods could significantly reduce SBP and DBP (102.26 ± 14.18 mmHg versus 112.86 ± 9.33 mmHg for SBP and 66.96 ± 10.39 mmHg versus 74.46 ± 7.61 mmHg for DBP) (*P* = 0.00 and *P* = 0.002, resp.). Also this study showed the night time BP was significantly lower in LGI period. This finding was in agreement with the results of a study by Philippou et al., who demonstrated that an LGI diet together with healthy eating and weight loss may be highly beneficial in reducing 24-hour BP [6]. Also, this finding was supported by previous studies, which demonstrated that LGI diets resulted in a significant reduction in SBP and DBP [8, 9].

Two interventional studies have demonstrated that lowering dietary GI decreases BP in adults [2, 10]. Furthermore, a previous systematic review and meta-analysis demonstrated that LGI diets can lower DBP in type 2 diabetic patients in the long run [11].

A reduction in postprandial plasma insulin may be of the mechanism which explains these changes. Insulin is known to activate the sympathetic nervous system and is a potential mediator of sodium retention and volume expansion, which result in higher BP [3].

We already demonstrated that increased dietary GI and GL lowered 24-hour SBP and DBP, although the difference was not statistically significant. These findings were supported by previous published data, showing that BP changes are insignificant after the HGI diet [6].

The results of the present study regarding the effect of HGI diet on BP were also confirmed by previous studies, which evaluated the effect of two hypocaloric LGI and HGI diets on obese children; the results demonstrated that both diets decreased BP in obese children [8, 9].

Conversely, a prospective study in 2004-2005 and 2009–2011, examining 858 students (aged 12 years), showed that increased intake of dietary CHO, specially HGI/HGL foods, could raise BP in females [3]; this finding was in contrast with the current results. However, it should be noted that this study had a large sample size, and participants were followed up for 5 years.

The current results showed a significant change in HR after the intervention in LGI and HGI groups during 24 hours. Also, Jenkins et al. demonstrated a significant difference in HR in their randomized controlled trial on 121 participants with type 2 diabetes mellitus after following both high wheat fiber and LGI diets [10].

To the best of our knowledge, this research is the first longitudinal study to examine the effect of dietary GI manipulation on 24-hour BP by adjusting the effects of confounding variables. Previous studies could not exclude confounding or unknown factors given the differences in subjects' characteristics such as age, weight, height, ethnicity, and BMI. Therefore, the possibility of selection bias, which limits the generalization of the results, could not be ruled out.

Also, in our study, the distribution of macronutrients and energy was similar in both diets. Power calculation using data from a study by Philippou et al. [6] and differences in SBP and DBP (differences of 3 and 13 mmHg) between the groups suggested a sample size of 30 subjects. It should be mentioned that the current study was performed over a short period of time; therefore, further longitudinal research is required for reaching a definite conclusion. Also, our study did not consider objective measurements such as biochemical factors and body composition.

## 5. Conclusion

In conclusion, the obtained results suggest that LGI foods may have significant reducing effects on SBP and DBP. Only the LGI group experienced a significant reduction in 24-hour BP, which may be related to the improvement in insulin sensitivity. However, other longitudinal studies with more comprehensive data are required to assess the relationship between BP, GI, and GL before reaching a definite conclusion.

## Figures and Tables

**Table 1:** Details and ingredients of consumed foods in 24-hour LGI period.

Food	Amount of intake	Carbohydrate (gr)	Protein (gr)	Fat (gr)	Calories (k cal)	%GI	GL (gr)
Special k	100 gr	79	9	1.5	375	54	11
Milk (3% fat)	500 cc	24	16	15	300	21	3
Oil	45 gr	0	0	45	405	0	0
Spaghetti	480 gr	192	12	0	800	42	20
Tomato sausage	30 gr	5	0	0	30	0	0
Total	—	**300**	**37**	**61.5**	**1910**		**34.1**
% of total calories		75.3%	9.3%	15.4%			

**Table 2:** Details and ingredients of consumed foods in 24-hour HGI period.

Food	Amount of intake	Carbohydrate (gr)	Protein (gr)	Fat (gr)	Calories (k cal)	%GI	GL (gr)
Corn flakes	50 gr	39.5	4.5	0.75	187.5	92	24
Milk (3% fat)	250 cc	12	8.25	7.5	150	21	3
Oil	45 gr	0	0	45	405	0	0
Rice	360 gr	162	9	0	660	84	45
Potato	300 gr	54	6	0	240	98	26
Tomato sausage	30 gr	5	0	0	30	0	0
Baguette	90 gr	66	9	0	240	108	24
Honey	30 gr	26	0	0	120	78	10
Total	—	**364.5**	**36.75**	**53.25**	**2032**		**132**
% of total calories		80%	8%	12%			

**Table 3:** Baseline characteristics of participants.

Variables	Percentage	Frequency
Age (years)		
18–20	10	3
20–25	53.3	16
25–30	33.3	10
30–35	3.3	1
BMI		
Underweight (BMI < 18.5 kg/m^2^)	6.7	2
Normal weight (BMI = 18.5–24.9 kg/m^2^)	83.3	25
Overweight (BMI = 25–29.9 kg/m^2^)	10	3
Obese (BMI > 30 kg/m^2^)	0	0

BMI: body mass index.

**Table 4:** The effects of LGI and HGI foods on SBP and DBP.

Characteristics	HGI diet			LGI diet		
	Baseline	At the end of the intervention	*P* value	Baseline	At the end of the intervention	*P* value
SBP (mmHg)	111.80 ± 9.57	110.66 ± 9.85	0.60	112.86 ± 9.33	102.26 ± 14.18	**0.001** ^*^
DBP (mmHg)	74.26 ± 10.09	71.16 ± 9.16	0.06	74.46 ± 7.61	66.96 ± 10.39	**0.002** ^*^
HR (bpm)	79.60 ± 12.77	71.13 ± 13.23	**0.01** ^*^	81.03 ± 12.71	72.23 ± 10.62	**0.02** ^*^

^∗^ *P* ≤ 0.05; intragroup comparison of baseline and after intervention (after 24 hours). Results were performed by paired *t*-test for normally distributed data and by Wilcoxon test for nonnormally distributed data.

**Table 5:** The effects of LGI and HGI foods on SBP and DBP divided by day and night.

	Characteristics	HGI diet			LGI diet		
		Baseline	At the end	*P* value	Baseline	At the end	*P* value
Day (10 am to 9 pm)	SBP (mmHg)	111.80 ± 9.57	139.1 ± 10.64	0.26	112.86 ± 9.33	112.43 ± 9.83	0.79
	DBP (mmHg)	74.26 ± 10.09	76.50 ± 8.43	0.16	74.46 ± 7.61	74.30 ± 8.99	0.91
	HR (bpm)	79.60 ± 12.77	77.13 ± 10.13	0.28	81.03 ± 12.71	76.63 ± 11.69	**0.007** ^*^

Night (10 pm to 8 am)	SBP (mmHg)	115.9 ± 9.96	110.66 ± 9.85	0.1	112.27 ± 10.62	102.26 ± 14.18	**0.01** ^*^
	DBP (mmHg)	74.10 ± 9.80	71.16 ± 9.16	0.65	74.30 ± 8.99	66.96 ± 10.39	**0.02** ^*^
	HR (bpm)	75.40 ± 17.53	71.13 ± 13.23	0.23	81.06 ± 11.87	72.23 ± 10.62	**0.001** ^*^

^∗^ *P* ≤ 0.05; intragroup comparison of baseline and after intervention (after 24 hours). Results were performed by paired *t*-test for normally distributed data and by Wilcoxon test for nonnormally distributed data.

**Table 6:** The comparison between percent changes of blood pressure in LGI foods and HGI foods.

	HGI diet	LGI diet	*P* value
Mean of 24-hour SBP (mmHg)	106.95 ± 6.34	106.39 ± 7.23	0.89
Mean of 24-hour DBP (mmHg)	69.50 ± 6.08	68.60 ± 5.8	0.31
Mean of 24-hour HR (bpm)	73.35 ± 8.72	74.03 ± 8.46	0.76
Changes in SBP after 24 hours (mmHg)	−1.13 ± 12.00	−10.60 ± 15.39	0.08
Changes in DBP after 24 hours (mmHg)	−3.10 ± 8.85	−7.50 ± 12.38	0.1
Changes in HR after 24 hours (bpm)	−8.40 ± 13.09	−8.8 ± 13.94	0.91
Changes in SBP during day (mmHg)	+2.10 ± 10.04	−4.33 ± 9.23	0.38
Changes in DBP during day (mmHg)	+2.23 ± 8.62	−0.16 ± 8.7	0.29
Changes in SBP during night (mmHg)	−3.23 ± 9.36	−10.16 ± 14.65	**0.01** ^*^
Changes in DBP during night (mmHg)	−2.93 ± 12.83	−8.76 ± 12.60	**0.04** ^*^
Changes in HR during day (bpm)	−2.46 ± 12.27	−4.40 ± 8.31	0.51
Changes in HR during night (bpm)	4.26 ± 19.17	8.83 ± 13.11	0.21

^∗^ *P* ≤ 0.05; within-group comparison of baseline and 24-hour results by paired *t*-test for normally distributed data or by Wilcoxon test for nonnormally distributed data.

## References

[1] Chen L., Caballero B., Mitchell D. C. (2010). Reducing consumption of sugar-sweetened beverages is associated with reduced blood pressure: a prospective study among United States Adults. *Circulation*.

[2] Appel L. J., Sacks F. M., Carey V. J. (2005). Effects of protein, monounsaturated fat, and carbohydrate intake on blood pressure and serum lipids: results of the OmniHeart randomized trial. *Journal of the American Medical Association*.

[3] Gopinath B., Flood V. M., Rochtchina E., Baur L. A., Smith W., Mitchell P. (2012). Influence of high glycemic index and glycemic load diets on blood pressure during adolescence. *Hypertension*.

[4] Hodgson J. M., Burke V., Beilin L. J., Puddey I. B. (2006). Partial substitution of carbohydrate intake with protein intake from lean red meat lowers blood pressure in hypertensive persons. *The American Journal of Clinical Nutrition*.

[5] Geleijnse J. M., Kok F. J., Grobbee D. E. (2004). Impact of dietary and lifestyle factors on the prevalence of hypertension in Western populations. *European Journal of Public Health*.

[6] Philippou E., Bovill-Taylor C., Rajkumar C. (2009). Preliminary report: the effect of a 6-month dietary glycemic index manipulation in addition to healthy eating advice and weight loss on arterial compliance and 24-hour ambulatory blood pressure in men: a pilot study. *Metabolism: Clinical and Experimental*.

[7] Foster-Powell K., Holt S. H. A., Brand-Miller J. C. (2002). International table of gylcemic index and glycemic load values: 2002. *The American Journal of Clinical Nutrition*.

[8] Parillo M., Licenziati M. R., Vacca M., de Marco D., Iannuzzi A. (2012). Metabolic changes after a hypocaloric, low-glycemic-index diet in obese children. *Journal of Endocrinological Investigation*.

[9] Iannuzzi A., Licenziati M. R., Vacca M. (2009). Comparison of two diets of varying glycemic index on carotid subclinical atherosclerosis in obese children. *Heart and Vessels*.

[10] Jenkins D. J. A., Kendall C. W. C., Augustin L. S. A. (2012). Effect of legumes as part of a low glycemic index diet on glycemic control and cardiovascular risk factors in type 2 diabetes mellitus: a randomized controlled trial. *Archives of Internal Medicine*.

[11] Mirrahimi A., Reiser E., Chiavaroli L. (2013). Low glycemic index diets on long-term blood pressure control: a systematic review and meta-analysis. *The FASEB Journal*.

